# Condensin minimizes topoisomerase II‐mediated entanglements of DNA *in vivo*

**DOI:** 10.15252/embj.2020105393

**Published:** 2020-11-06

**Authors:** Sílvia Dyson, Joana Segura, Belén Martínez‐García, Antonio Valdés, Joaquim Roca

**Affiliations:** ^1^ Molecular Biology Institute of Barcelona (IBMB) Spanish National Research Council (CSIC) Barcelona Spain

**Keywords:** chromatin, DNA knot, DNA loop extrusion, DNA topology, SMC complex, Chromatin, Epigenetics, Genomics & Functional Genomics, DNA Replication, Repair & Recombination

## Abstract

The juxtaposition of intracellular DNA segments, together with the DNA‐passage activity of topoisomerase II, leads to the formation of DNA knots and interlinks, which jeopardize chromatin structure and gene expression. Recent studies in budding yeast have shown that some mechanism minimizes the knotting probability of intracellular DNA. Here, we tested whether this is achieved via the intrinsic capacity of topoisomerase II for simplifying the equilibrium topology of DNA; or whether it is mediated by SMC (structural maintenance of chromosomes) protein complexes like condensin or cohesin, whose capacity to extrude DNA loops could enforce dissolution of DNA knots by topoisomerase II. We show that the low knotting probability of DNA does not depend on the simplification capacity of topoisomerase II nor on the activities of cohesin or Smc5/6 complexes. However, inactivation of condensin increases the occurrence of DNA knots throughout the cell cycle. These results suggest an *in vivo* role for the DNA loop extrusion activity of condensin and may explain why condensin disruption produces a variety of alterations in interphase chromatin, in addition to persistent sister chromatid interlinks in mitotic chromatin.

## Graphical abstract

Preventing knotting and interlinking of juxtaposed DNA segments requires yeast condensin but not cohesin or Smc5/6 complexes, suggesting an *in vivo* role for the DNA loop‐extrusion activity of this particular SMC protein complex.

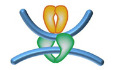

## Introduction

Type‐2A topoisomerases, such as bacterial topo IV and eukaryotic topo II, pass one segment of duplex DNA through the transient double‐stranded DNA break that they produce in another segment (Wang, 1998). This DNA‐passage activity is essential to remove the intertwines generated between newly replicated DNA molecules and to modulate DNA supercoiling during genome transactions (Corbett & Berger, 2004; Nitiss, 2009). However, the activity of type‐2A topoisomerases also entails important threats. Firstly, the DNA cleaving step can be a source of chromosomal damage (Nitiss & Wang, 1996). Secondly, the DNA‐passage activity can entangle DNA molecules that are closely packed or folded via protein‐DNA interactions (Hsieh, 1983; Wasserman & Cozzarelli, 1991; Roca et al, 1993). Indeed, computer simulations have predicted that DNA molecules confined in biological systems would be massively entangled if type‐2A topoisomerases could freely equilibrate their global topology (Arsuaga et al, 2002; Micheletti et al, 2008; Dorier & Stasiak, 2009). Fortunately, this prospect does not occur because the hierarchical folding of chromatin, which has a scaling behavior similar to that of a fractal globule, drastically reduces the topological complexity of chromosomes (Lieberman‐Aiden et al, 2009; Mirny, 2011). Accordingly, 3D analyses of the eukaryotic nuclear architecture revealed little intermingling of chromosomal territories and large chromatin domains (Denker & de Laat, 2016; Schmitt et al, 2016). Reconstruction of 3D paths of high‐order chromatin fibers in individual cells also evidenced the scarcity of long‐range entanglements (Siebert et al, 2017, Stevens et al, 2017; Sulkowska et al, 2018).

**Figure 1:**
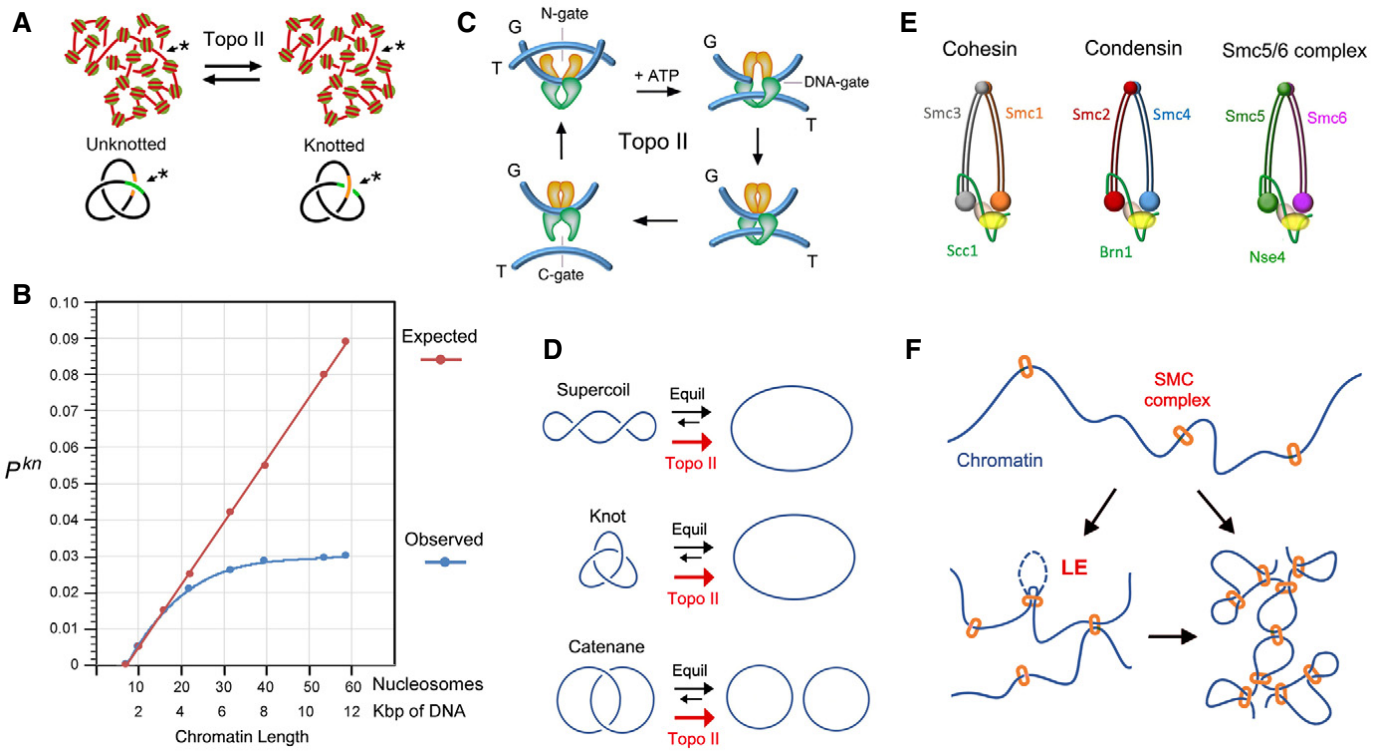
Knotting probability of intracellular DNA and plausible regulatory mechanisms Topo II activity on random juxtapositions of DNA segments (*) produces steady‐state fractions of DNA knots in intracellular chromatin.DNA knotting probability (*P^kn^*) of intracellular chromatin (observed) does not scale proportionally to the length of DNA (expected). The slope of *P^kn^* is reduced in chromatin stretches larger than 20 nucleosomes. Data from (Valdes et al, 2018).Three‐gate mechanism of topo II to pass one segment of DNA (T‐segment) through another (G‐segment). Upon ATP binding, the T‐segment is captured by the entrance gate (N‐gate) and passed through the transiently cleaved G‐segment (DNA‐gate). Upon re‐ligation of the G‐segment, the T‐segment is released through the exit gate (C‐gate).Topo II activity reduces the fractions of DNA supercoils, knots and catenates to below the topological equilibrium values (see details in Fig EV1).Architecture of the SMC complexes of *S*. *cerevisiae*. The Smc heterodimers (Smc1‐Smc3, Smc2‐Smc4, Smc5‐Smc6) and kleisin (Scc1, Brn1, Nse4) subunits of cohesin, condensin, and the Smc5/6 complex are indicated.SMC complexes entrap segments of DNA to form chromatin loops and/or bridge nearby chromatin domains. Their loop extrusion activity (LE) ensures the co‐entrapment of contiguously oriented intramolecular DNA segments.

The hierarchical architecture of chromatin, however, cannot prevent the formation of DNA interlinks between chromatin fibers that come in close proximity or the formation of DNA knots within clusters of nucleosomes. Another mechanism must thereby operate to avoid these local DNA entanglements. This is exemplified by the sister chromatid interlinks (SCI), which are eliminated during prophase, even though sister chromatids remain cohesed until metaphase (Nagasaka et al, 2016). Further evidence of such mechanism also emerged from the analysis of the knotting probability (*P^kn^*) of intracellular chromatin (Valdes et al, 2018). These studies revealed that topo II‐mediated knotting and unknotting of DNA normally occur within stretches of 10 to 60 nucleosomes (Fig 1A). However, the *P^kn^* of chromatin does not scale proportionately to DNA length, as would be expected for any polymer chain. The slope of *P^kn^* is progressively reduced in domains larger than 20 nucleosomes (Valdes et al, 2018), as if some mechanism were counteracting the potential entanglement of intracellular DNA (Fig 1B).

**Figure EV1:**
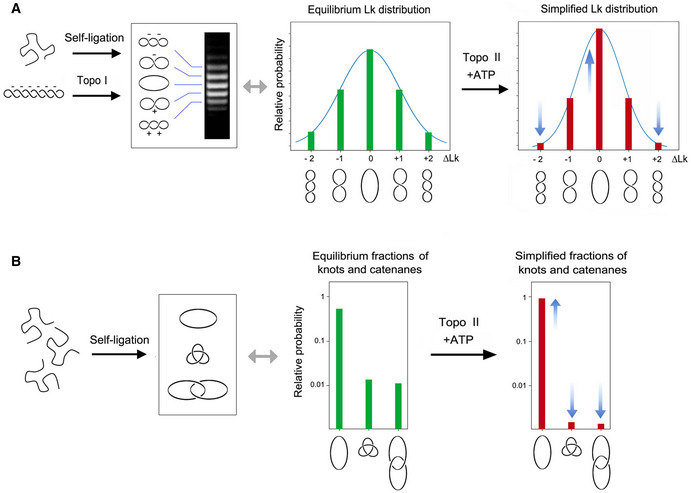
Equilibrium DNA topology and its simplification by Topo II Either the self ligation of a linear DNA duplex into a covalently closed ring or the relaxation of a DNA plasmid with a type‐1B topoisomerase (topo I) produce identical equilibrium distributions of Lk topoisomers, which reflect the thermal fluctuations (twisting and bending) of DNA molecules in free solution. ATP‐dependent DNA passage catalyzed by topo II simplifies (i.e., reduces the variance, narrows) the equilibrium distribution of Lk topoisomers. ∆Lk values indicate the Lk difference relative to the distribution center (Lk = 0).Circularization of linear DNA molecules in free solution can also produce knotted and/or catenated DNA rings. Knotting probability increases with DNA length, whereas catenane probability increases with DNA concentration. As in the case of the Lk distribution, the knotting and catenation probability reflect the equilibrium topology of DNA in free solution. ATP‐dependent DNA passage catalyzed by topo II markedly reduces (i.e., simplifies) the equilibrium fractions of knotted and catenated forms.

**Figure EV2:**
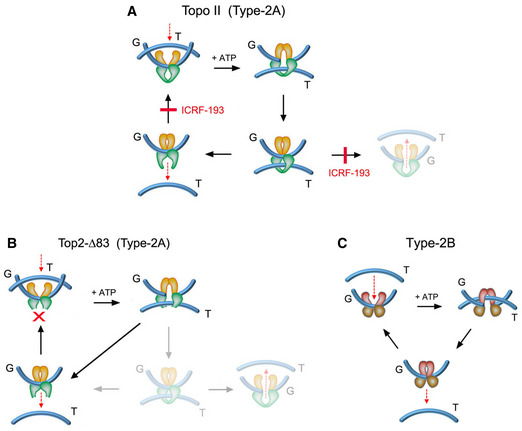
Conditions that preclude topo II capacity to simplify DNA topology The topo II inhibitor ICRF‐193 impedes the reopening of the N‐gate once the T‐segment has been captured and passed across the G‐segment. ICRF‐193 blocks thereby the enzyme turnover and the plausible backtracking of the T‐segment across the G‐segment. When topo II activity is quenched with ICRF‐193, the last DNA‐passage event conducted by the enzyme does not simplify the equilibrium DNA topology.The topo II construct top2‐∆83, in which the C‐gate has been deleted, is able to perform DNA passage and the T‐segment cannot backtrack since it is freed upon crossing the G‐segment. This truncated enzyme can relax and unlink DNA molecules but has lost the capacity to simplify the equilibrium DNA topology.Type‐2B topoisomerases are mechanistically similar to type‐2A topoisomerases (topo II). The T‐segment is captured by the N‐gate and is passed across the bended G‐segment at the DNA‐gate. However, type‐2B topoisomerases do not have a C‐gate, so the passed T‐segment is naturally freed upon crossing the G‐segment. As in the case of top2‐∆83, type‐2B topoisomerases relax and unlink DNA molecules but do not simplify equilibrium DNA topology.

Two mechanisms have been hypothesized that could minimize the entanglement of DNA *in vivo*. The first one relies on the intrinsic capacity of type‐2A topoisomerases to simplify the equilibrium topology of DNA molecules in free solution (Rybenkov et al, 1997). Namely, topo II uses a three‐gate mechanism to pass one segment of DNA (T‐segment) through another (G‐segment) in an ATP‐dependent manner (Wang, 1998). Upon topo II binding to the G‐segment, the T‐segment is captured by closing the entrance gate (N‐gate) of the enzyme. The T‐segment is then passed through the transiently cleaved G‐segment (DNA‐gate), and it is released outside the enzyme through the exit gate (C‐gate) (Fig 1C). This mechanism allows DNA supercoils, knots, and catenates to be reduced. However, when topo II relaxes supercoiled DNA, it produces a linking number (Lk) distribution of DNA topoisomers that is narrower than the equilibrium Lk distribution (Figs 1, EV1 and 1, EV1). Likewise, when topo II unknots or decatenates DNA molecules, it reduces the fraction of knotted and catenated molecules to values below the topological equilibrium (Figs 1, EV1). The mechanism by which topo II is able to assess and locally reduce the equilibrium topology of large DNA molecules remains mysterious (Vologodskii, 2016). Moreover, the physiological relevance of this simplification activity is unknown since it has never been assessed *in vivo*. Yet, *in vitro* studies have shown that topo II does not simplify the equilibrium topology of DNA when the enzyme activity is quenched with the inhibitor ICRF‐193 (Fig EV2A) or when the C‐gate of the enzyme is deleted (Fig EV2B) (Martinez‐Garcia et al, 2014; Thomson et al, 2014). These two observations opened up the possibility to target the simplification activity of intracellular topo II and test whether that affects the *P^kn^* of chromatin.

The second mechanism that could reduce intracellular DNA entanglements relies on the activity of structural maintenance of chromosomes (SMC) complexes, which are mainly identified as cohesin, condensin, and the Smc5/6 complex in eukaryotic cells (Uhlmann, 2016; Yatskevich et al, 2019). Cohesin generates the DNA loops that organize chromatin during interphase and holds sister chromatids together from S‐phase until metaphase (Onn et al, 2008; Nasmyth & Haering, 2009). Condensin plays a key role in the compaction and individualization of chromatids during cell divisions (Hirano, 2012). The Smc5/6 complex has been mainly implicated in DNA repair via homologous recombination (Aragon, 2018). Despite their distinct roles, SMCs have similar architecture. They are large rod‐shaped proteinic ensembles composed of a trimeric core formed by a heterodimer of Smc ATPases and a conserved kleisin, in addition to several additional regulatory subunits (Fig 1E). ATP binding and hydrolysis produce the opening and closure of distinct SMC compartments, which can embrace one or more segments of DNA (Hassler et al, 2018; Yatskevich et al, 2019). Moreover, ATP usage can produce the translocation of the SMC complex along DNA (Terakawa et al, 2017) and the extrusion of DNA loops (Ganji et al, 2018; Davidson et al, 2019; Kim et al, 2019) (Fig 1F). The notion that SMCs can promote the removal of DNA entanglements was proposed in the context of sister chromatid resolution (Sen et al, 2016; Piskadlo et al, 2017). Former studies suggested that positive supercoils generated by condensin in mitotic chromosomes could produce a bias in topo II activity to eliminate the SCIs (Baxter et al, 2011; Sen et al, 2016). Subsequent computational simulations showed that DNA loop extrusion activity of SMCs would constrict DNA entanglements and so bias topo II to disentangle intermixed chromosomes (Goloborodko et al, 2016a) and minimize the occurrence of DNA knots (Racko et al, 2018; Orlandini et al, 2019).

Here, we show that precluding the capacity of topo II to simplify equilibrium topology of DNA does not alter the low knotting probability of intracellular chromatin. Inactivation of cohesin or the smc5/6 complex also does not increase knot formation. However, inactivation of condensin markedly increases the occurrence of chromatin knots throughout the cell cycle. We propose that the requirement of condensin to minimize DNA entanglements might rely on its DNA loop extrusion activity. This function could explain the wide range of alterations that condensin inactivation produces both in interphase and mitotic chromatin.

## Results

### Topoisomerase II does not minimize the knotting probability of chromatin

**Figure 2:**
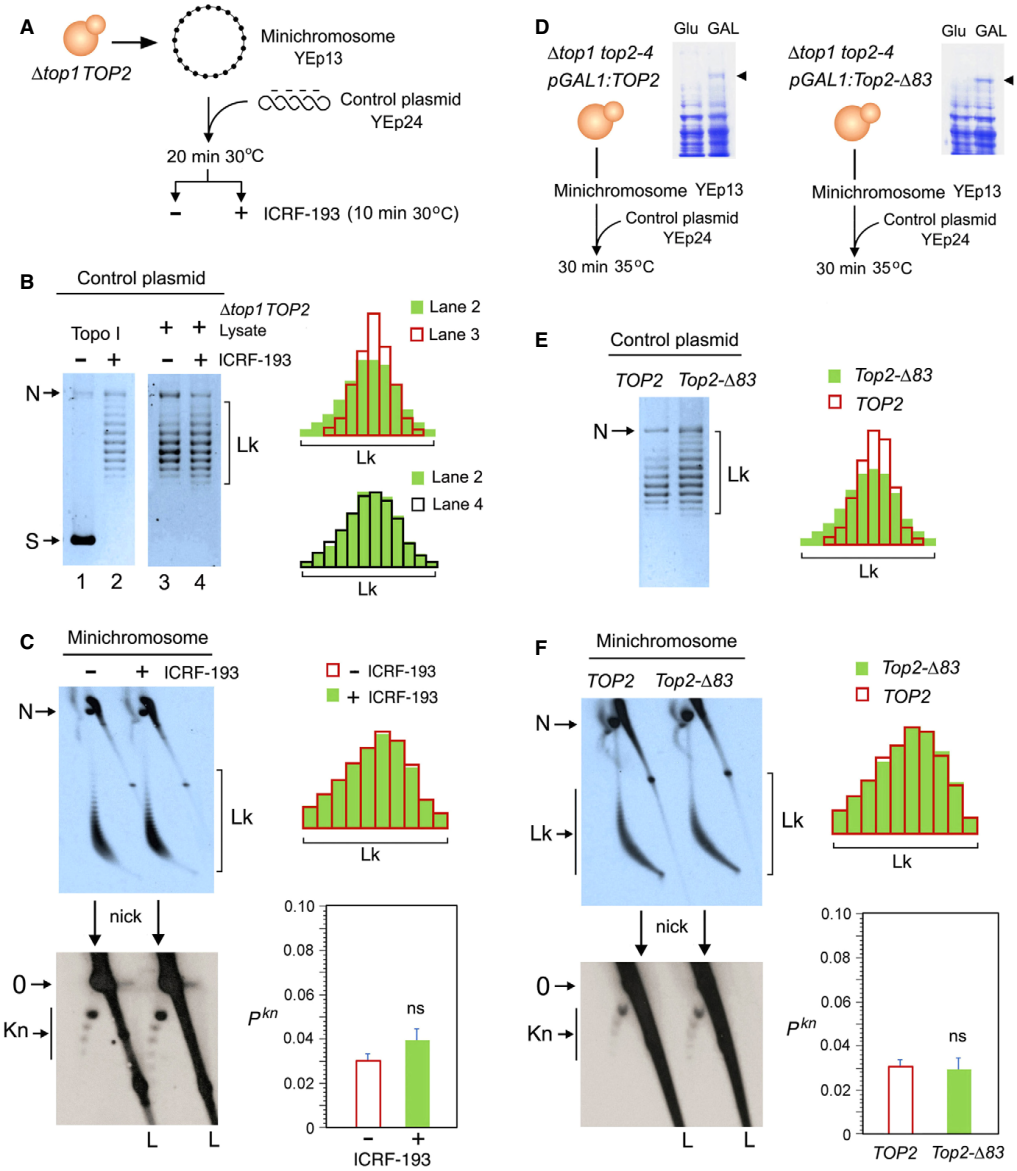
Topoisomerase II does not minimize the knotting probability of chromatin AExperimental layout to test the DNA topology simplification activity of cellular topo II upon the addition of ICRF‐193.BLanes 1 and 2: negatively supercoiled plasmid (YEp24) and its equilibrium distribution of Lk topoisomers upon its relaxation with Topo I. Lanes 3 and 4: distribution of Lk topoisomers of YEp24 upon its relaxation in lysates of *∆top1 TOP2* yeast cells in absence and after the addition of ICRF‐193. Plots compare the relative intensity of individual topoisomers of the Lk distributions in lanes 2, 3, and 4.CTop: 2D gel electrophoresis of the Lk distributions of the YEp13 minichromosome present in the lysates of *∆top1 TOP2* yeast cells before and after the addition of ICRF‐193 (see details in Fig EV3). Plots compare the relative intensity of the Lk distributions (divided into ten sections). Bottom: 2D gel electrophoresis of the same samples upon nicking the DNA in order to reveal the occurrence of knots (see details in Fig EV4). The graph shows *P^kn^* of YEp13 (mean ± SD from three independent experiments). P‐values (Student’s t test): ns, *P *> 0.05.DExperimental layout to compare the activities of *TOP2* and *Top2‐∆83* on DNA and chromatin. Arrowheads indicate the extrachromosomal expression of *TOP2* and *Top2‐∆83* under the inducible *pGAL1* promoter.ELk distributions of the control plasmid (YEp24) relaxed by lysates of *∆top1 top2‐4* yeast cells that expressed *TOP2* or *Top2‐∆83*. Plots compare the relative intensity of individual Lk topoisomers.FTop: 2D gel electrophoresis of the Lk distributions of the YEp13 minichromosome produced in the presence of *TOP2* or *Top2‐∆83*. Plots compare the relative intensity of the Lk distributions (divided into ten sections). Bottom: 2D gel electrophoresis of the same samples upon nicking the DNA in order to reveal the occurrence of knots. Graph: *P^kn^* of YEp13 (mean ± SD from three independent experiments). Gel signals are: N, nicked DNA circles; S, supercoiled DNA; L, diagonal of linear DNA fragments; Lk, distribution of Lk topoisomers; 0, unknotted DNA (nicked); Kn, ladder of knotted forms (nicked). P‐values (Student’s *t* test): ns, *P* > 0.05.

**Figure EV3:**
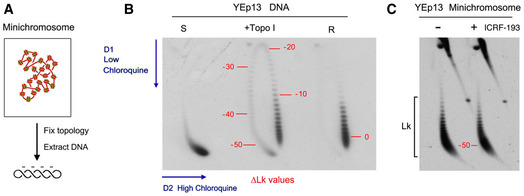
2D gel electrophoresis of the DNA linking number distribution of circular minichromosomes DNA molecules extracted from yeast circular minichromosomes are negatively supercoiled since each nucleosome constrains about one negative supercoil (∆Lk ≈ −1).2D gel electrophoresis of covalently closed DNA circles, in which the first and second gel dimensions are run in the presence of low and high concentrations of chloroquine, respectively, allow the Lk distribution of DNA topoisomers to be resolved along an arch, in which Lk values increase clockwise. The 2D gel shows highly negatively supercoiled (S), partially relaxed (+topo I), and fully relaxed (R) forms of the YEp13 plasmid. Numbers in red indicate approximate ∆Lk values relative to the center of the relaxed (R) Lk distribution (∆Lk = 0).2D gel electrophoresis of DNA of the YEp13 minichromosome (as in Fig 2C). Comparison of the gels in B and C indicates that DNA in the YEp13 minichromosome is negatively supercoiled and has ∆Lk close to −50. This value is consistent with the plausible number of nucleosomes assembled in YEp13 (10.7 Kb). However, note that since the outline of Lk distributions can vary in separate 2D gels (i.e., due to differences in tank dimensions, power supply and temperature during electrophoresis), only DNA samples that ran in the same gel (side by side) can be accurately compared.

**Figure EV4:**
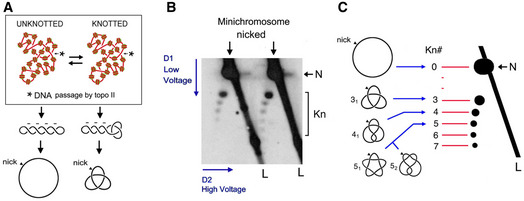
2D gel electrophoresis of DNA knots formed in yeast circular minichromosomes DNA molecules extracted from yeast minichromosomes might contain knots due to the knotting‐unknotting activity of intracellular topo II. Knotted and unknotted molecules are hard to distinguish when DNA is supercoiled because all of them present similar compaction. Upon nicking the DNA, supercoiling is dissipated and knotted molecules remain more compact than unknotted ones.2D gel electrophoresis of nicked DNA of the YEp13 minichromosome (as in Fig 2C). The first and second gel‐dimensions run at low and high voltage, respectively. In the first dimension, knotted molecules (Kn) are more compacted and so move faster than unknotted ones (N). In the second gel‐dimension, knotted molecules are retarded from the diagonal of linear DNA fragments (L), which produces a strong signal due to genomic DNA present in the samples.Identification of DNA knot populations according to the irreducible number of DNA crossings of each knot (Kn#). From the position of the unknotted circle that has zero crossings (0), a ladder of knot populations of increasing complexity begins with the knot of three crossings (3_1_), followed by the knot with four crossing (4_1_), two knots with five crossings (5_1_ and 5_2_), and so on.

We used two experimental approaches to assess whether the capacity of topo II to simplify the equilibrium topology of DNA was sustaining the low knotting probability (*P^kn^*) of intracellular chromatin. First, we used the topo II inhibitor ICRF‐193 to impair the simplification activity of cellular topo II (Fig EV2A) (Martinez‐Garcia et al, 2014). We carried out this experiment in a *∆top1 TOP2* yeast strain that hosted the circular minichromosome YEp13 (10.7 Kb) as the reporter of *P^kn^* (Fig 2A). To verify that the simplification capacity of yeast topo II was targeted by ICRF‐193, we added to crude lysates of the cells a negatively supercoiled DNA plasmid (YEp24, 7.8 Kb), which served as internal control of topo II activity (Fig 2A). When YEp24 was relaxed by a purified type‐1B topoisomerase (topo I) (Fig 2B, lanes 1 and 2), it produced an equilibrium distribution of Lk topoisomers (Fig EV1A). However, when YEp24 was relaxed by the topo II activity present in the cell lysates, it produced a distribution of Lk topoisomers that was narrower than the equilibrium Lk distribution generated by topo I (Fig 2B, compare Lk plots of lanes 2 and 3). When we quenched the topo II activity by adding ICRF‐193 to the mixture, the Lk distribution of YEp24 became broadened to an extent similar to that of the equilibrium Lk distribution (Fig 2B, compare Lk plots of lanes 2 and 4). Therefore, cellular topo II was able to simplify the equilibrium topology of naked DNA and this capacity was canceled by ICRF‐193. We then examined what happened to the topology of the YEp13 minichromosome present in the above mixtures before and after the addition of ICRF‐193 (Fig 2C). To this end, we first ran a 2D‐gel electrophoresis containing chloroquine (Hanai & Roca, 1999) to resolve the Lk distribution of the YEp13 DNA (Fig EV3). Before adding ICRF‐193, YEp13 presented a distribution of topoisomers of negative ∆Lk values (Fig 2C, Lk) consistent with the negative supercoils constrained by nucleosomes (Segura et al, 2018) (Fig EV3). Following the addition of ICRF‐193, the Lk distribution of YEp13 was not significantly altered, which contrasted to what was observed in the control plasmid YEp24 (compare Lk plots in Fig 2C and 2B). Next, we nicked the DNA samples and ran a different 2D gel electrophoresis to reveal DNA knots (Trigueros et al, 2001)(Fig EV4). Both before and after adding ICRF‐193, YEp13 presented a similar ladder of knotted molecules (Fig 2C, Kn), which started with the knot of three irreducible crossings (trefoil knot or 3_1_) as the most abundant form (Fig EV4). We calculated *P^Kn^* as the relative abundance of total knotted molecules with respect to the total amount of unknotted and knotted DNA circles. In agreement with previous studies, the *P^kn^* of YEp13 was about 0.03 (Valdes et al, 2018), three times lower than the value expected if *P^kn^* scaled proportionally to DNA length (Fig 1B). Following the addition of ICRF‐193, the *P^kn^* of YEp13 was not significantly altered (Fig 2C).

Our second approach to test the simplification activity of topo II on chromatin was via the expression of a truncated topo II (*Top2‐∆83*), in which the C‐gate was removed (Martinez‐Garcia et al, 2014) (Fig 2D, Fig EV2B). Similar to the type‐2B class of topoisomerases that innately lack a C‐gate (Fig EV2C), *Top2‐∆83* is able to reduce DNA topology constraints but cannot simplify the equilibrium topology of free DNA (Martinez‐Garcia et al, 2014; Thomson et al, 2014). We used the galactose‐inducible *pGal1* promoter to express in *∆top1 top2‐4* yeast cells either *Top2‐∆83* or *TOP2* (full‐length topo II) enzymes (Fig 2D). Upon inactivation of the *top2‐4* thermo‐sensitive allele, we examined the effects of the expressed enzymes on the topology of the control plasmid YEp24 and the minichromosome Yep13 present in these cells. As expected, in cells expressing *TOP2*, YEp24 was relaxed and presented a narrow (i.e., simplified) distribution of Lk topoisomers, whereas in cells expressing *Top2‐∆83*, the resulting Lk distribution was wider (Fig 2E). However, neither the Lk distribution nor the knotting probability of the Yep13 minichromosome was altered in the presence of the *TOP2* and *Top2‐∆83* activities (Fig 2F). Therefore, in concordance with the ICRF‐193 results, precluding the simplification capacity of topo II did not produce any significant effect on the topology of chromatinized DNA.

### Condensin inactivation boosts the occurrence of chromatin knots

To test whether the low knotting probability of intracellular DNA was achieved via the activity of SMC complexes, we examined the DNA topology of the Yep13 minichromosome in yeast strains previously characterized for carrying thermo‐sensitive mutations that inactivate either condensin (*smc2‐8*) (Freeman et al, 2000), cohesin (*scc1–73*)(Michaelis et al, 1997), or the Smc5/6 complex (*smc6‐9*) (Torres‐Rosell et al, 2005) (Appendix Fig S2). In each case, we grew the cells at a permissive temperature (26°C) and, upon reaching the exponential phase (OD 0.6‐0.8), we shifted one half of the cultures to 35°C for 60 min. We then fixed the topology of intracellular DNA by quenching the cells with a cold ethanol‐toluene solution and extracted their total DNA (Diaz‐Ingelmo et al, 2015). As in the foregoing experiments, we ran a 2D‐gel electrophoresis to examine the distribution of Lk topoisomers of YEp13; and we nicked the DNA to examine the occurrence of DNA knots in a different 2D gel electrophoresis.

**Figure 3:**
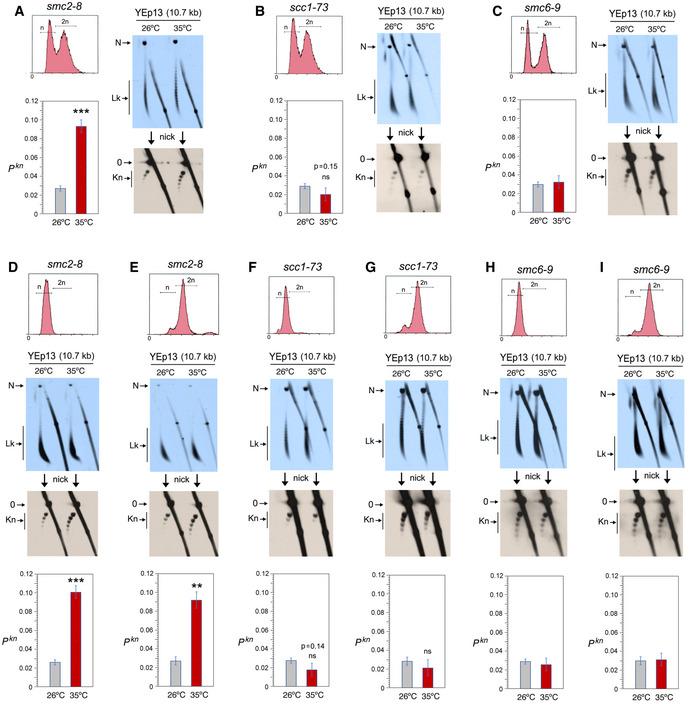
Condensin inactivation boosts the occurrence of chromatin knots ATop, DNA content (n/2n) of exponentially growing (OD_600_ = 0.6–0.8) *smc2‐8* yeast cells. First blot: 2D gel electrophoresis of the distribution of Lk topoisomers (Lk) of the YEp13 DNA in cells quenched at 26°C and after shifting the culture to 35°C for 60 min. Second blot: 2D gel electrophoresis of the same samples upon nicking the YEp13 DNA in order to reveal the occurrence of knots (kn). Graph: *P^kn^* of YEp13 before and after the inactivation of condensin.BExperiments conducted as in (A) but in *scc1‐73* yeast cells. Graph: *P^kn^* of YEp13 before and after the inactivation of cohesin.CExperiments conducted as in (A) but in *smc6‐9* yeast cells. Graph: *P^kn^* of YEp13 before and after the inactivation of the Smc5/6 complex.D, EExperiments conducted as in (A), but in cells arrested in G_1_ with alpha‐factor (D) or in metaphase with nocodazole (E) for 2 h at 26°C and for one additional hour at 26°C or 35°C.F, GExperiments conducted as in (B), but in cells arrested in G_1_ with alpha‐factor for 2 h at 26°C (F) or in metaphase with nocodazole (G) and for one additional hour at 26°C or 35°C.H, IExperiments conducted as in (C), but in cells arrested in G_1_ with alpha‐factor (H) or in metaphase with nocodazole (I) for 2 h at 26°C and for one additional hour at 26°C or 35°C. Data information: Gel signals (N, Lk, 0, Kn) are as described in Fig 2. Graphs show mean ± SD from three independent experiments in (A, B, D, E, F, G); and from two independent experiments in (C, H, I). *P*‐values (Student’s t test): ns, *P* > 0.05; **P < 0.01; ***P < 0.001.

**Figure EV5:**
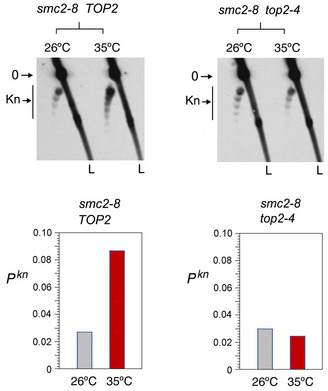
Topo II dependence of knot formation upon inactivation of condensin The *smc2‐8* mutation was introduced by gene replacement in yeast strains JCW25 (*TOP2*) and JCW26 (*top2‐4*). The 2D gel electrophoresis shows the knotted forms of the minichromosome YEp13 in the resulting *smc2‐8 TOP2* and *smc2‐8 top2‐4* mutants sampled at 26°C and after shifting the cell cultures to 35°C for 60 min. Gel signals: 0, unknotted DNA circles; Kn, knotted forms. L, linear DNA fragments. Graphs: *P^kn^* of YEp13 before and after inactivation of the thermo‐sensitive alleles.

The Lk distribution of YEp13 in the three strains presented negative ∆Lk values, which were not significantly altered upon inactivation of condensin, cohesin, or the Smc5/6 complex (Fig 3A‐C). Likewise, before inactivation of the SMCs, the knotting probability of YEp13 was low and similar in the three strains (*P^kn^*≈0.03) (Fig 3A‐C). This concordance indicated that the knot minimization mechanism is robust and performs equally in most cells. However, upon inactivation of condensin, *P^kn^* of YEp13 increased about threefold (*P^kn^* ≈0.09) (Fig 3A). Inactivation of cohesin produced a slight yet not significant reduction (*P^kn^* ≈0.02) (Fig 3B). Inactivation of the Smc5/6 complex did not change the knot abundance (Fig 3C). To verify that the *smc2‐8* allele was causing the threefold increase of *P^kn^*, we introduced this mutation in strains JCW25 (*TOP2*) and JCW26 (*top2‐4*) (Trigueros & Roca, 2001). Upon shifting these cells to 35°C, the *P^kn^* of YEp13 increased again about threefold in the *smc2‐8 TOP2* cells (Fig EV5). However, DNA knot formation did not change in the *smc2‐8 top2‐4* double mutant, which corroborated that topo II activity is required to produce the *P^kn^* changes induced by condensin (Fig EV5).

Since the above experiments were done in asynchronous cell cultures, we considered whether the effects of SMC inactivation on *P^kn^* would occur at different stages of the cell cycle. We conducted analogous experiments in cells arrested in G_1_ and in metaphase (Fig 3D‐3I). Arrested cells were sampled at 26°C and after shifting them to 35°C for 60 min during the arrest. Prior inactivation of the SMCs, *P^kn^* of YEp13 in the G_1_ and the metaphase‐arrested cells were similar to that observed in the asynchronous cell cultures (P^kn^≈0.03) (Fig 3D‐3I). This observation corroborated previous indications that the knotting probability of intracellular chromatin is not cell cycle‐dependent (Valdes et al, 2018). Upon inactivation of condensin, the occurrence of knots in YEp13 increased about threefold both in G_1_ (*P^kn^* ≈0.10) and in metaphase‐arrested cells (*P^kn^* ≈0.09) (Fig 3D and 3E). Inactivation of cohesin produced a slight reduction of *P^kn^* in G_1_ (P^kn^ ≈0.017) and metaphase cells (*P^kn^* ≈0.022) (Fig 3F and 3G). Inactivation of the Smc5/6 complex did not alter the knot abundance at any stage (Fig 3H and 3I). Thus, we concluded that inactivation of condensin markedly increases the occurrence of DNA knots throughout the cell cycle. Remarkably, this change of *P^kn^* occurred without any notable alteration of the Lk distribution of the minichromosome. Therefore, the regulation of *P^kn^* by condensin did not involve changes of DNA supercoiling or a major disruption of chromatin structure.

### Condensin inactivation restores the DNA length‐dependent entanglement of chromatin

Next, we asked whether the effects of condensin, cohesin, and Smc5/6 activity on the knotting probability of YEp13 were reproduced in other chromatin constructs. To this end, we transformed the SMCs mutant strains with circular minichromosomes that contained distinct functional elements (replication origins, transcription units, centromeres) and differed in DNA length. We inspected the topology of minichromosomes YRp3 (3.2 kb), YRp4 (4.4 kb), YRp5 (5.0 kb), YCp50 (7.9 kb), YRp21(11.7 Kb), and of the endogenous 2‐micron plasmid (6.3 kb) present in yeast cells (Appendix Fig S1). As in the foregoing experiments, we sampled exponentially growing cultures before and after inactivation of the SMCs.

**Figure 4:**
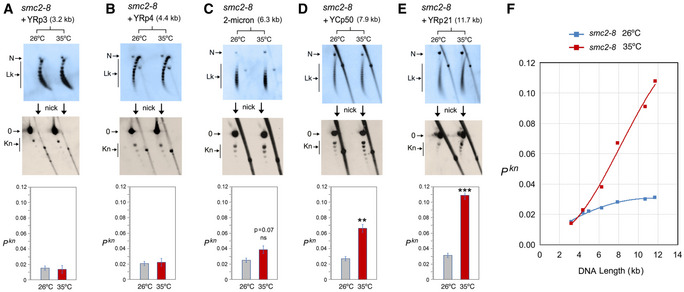
DNA length dependence of the topological effects of condensin inactivation A–EDNA topology of the indicated minichromosomes of increasing DNA length (kb) before (26°C) and after inactivation of condensin (35°C) in *smc2‐8* cells. In each case, the first 2D gel resolves the Lk topoisomers (Lk), the second 2D gel uncovers the knotted forms (Kn). Gel signals are denoted as in Fig 2. Bottom graphs compare the *P^kn^* before and after the inactivation of condensin (mean ± SD from three independent experiments). P‐values (Student’s t test): ns, p> 0.05; **p < 0.01; ***p < 0.001.FPlot of *P^kn^* values of minichromosomes of increasing DNA length (including YEp13) before and after inactivation of condensin.

In the *smc2‐8* mutant (Fig 4), condensin inactivation did not significantly change the knot probability of YRp3, YRp4, and the 2‐micron plasmid (Fig 4A–C). However, it augmented the occurrence of knots about twofold in YCp50 (Fig 4D), and over threefold in YRp21 (Fig 4E). Therefore, the effect of condensin inactivation on *P^kn^* values appeared to vary with DNA length rather than with the presence of specific functional elements. Furthermore, plotting the *P^kn^* changes of the distinct minichromosomes revealed that the inactivation of condensin increased *P^kn^* to the levels expected if knot formation was to escalate proportionally to DNA length (Fig 4F).

**Figure 5:**
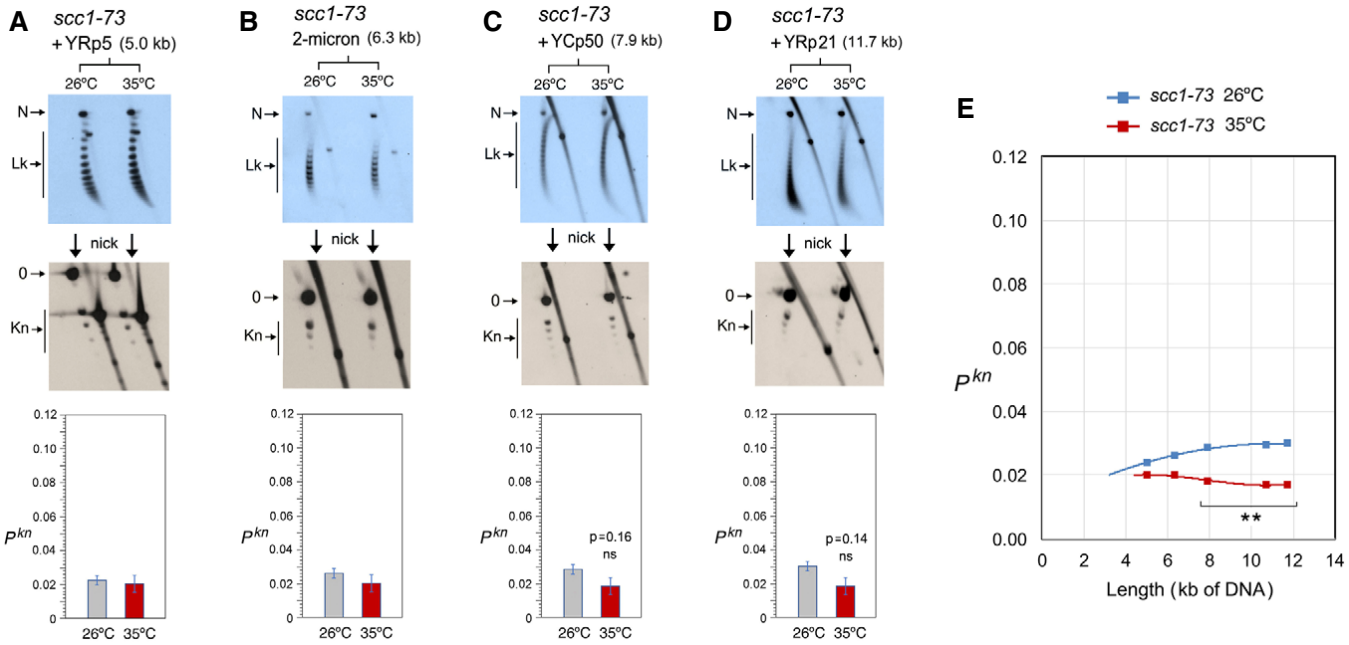
DNA length dependence of the topological effects of cohesin inactivation A–DDNA topology of the indicated minichromosomes of increasing DNA length (kb) before (26°C) and after inactivation of cohesin (35°C) in *scc1‐73* cells. In each case, the first 2D gel resolves the Lk topoisomers (Lk), the second 2D gel uncovers the knotted forms (Kn). Gel signals are denoted as in Fig 2. Bottom graphs compare the *P^kn^* before and after the inactivation of cohesin (mean ± SD from three independent experiments). *P*‐values (Student’s *t* test): ns, *P* > 0.05.EPlot of *P^kn^* values of minichromosomes of increasing DNA length (including YEp13) before and after inactivation of cohesin. *P*‐values (Student’s *t* test): **P < 0.01.

**Figure 6:**
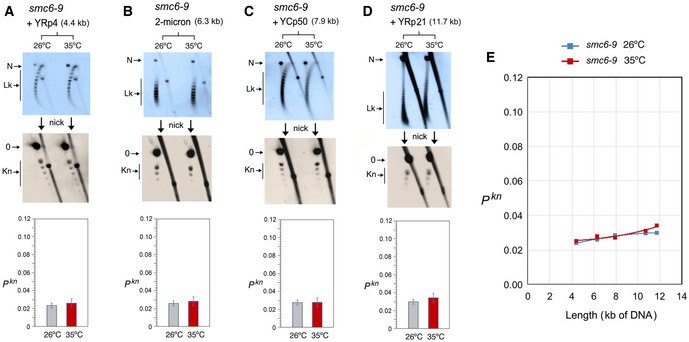
DNA length dependence of the topological effects of Smc5/6 complex inactivation A–DDNA topology of the indicated minichromosomes of increasing DNA length (kb) before (26°C) and after inactivation of Smc5/6 complex (35°C) in *smc6‐9* cells. In each case, the first 2D gel resolves the Lk topoisomers (Lk), and the second 2D gel uncovers the knotted forms (Kn). Gel signals are denoted as in Fig 2. Bottom graphs compare the *P^kn^* before and after the inactivation of Smc5/6 complex (mean ± SD from two independent experiments).EPlot of *P^kn^* values of minichromosomes of increasing DNA length (including YEp13) before and after inactivation of Smc5/6 complex.

In the *scc1–73* mutant (Fig 5), YRp3 and YRp4 could not be analyzed since the strain was *TRP+*. Cohesin inactivation did not change the knot probability of YRp5 and the 2‐micron plasmid (Fig 5A and B). However, similar to that observed in YEp13 (Fig 3B), cohesin inactivation produced a slight reduction of *P^kn^* in YCp50 and YRp21 (Fig 5C andD). Plotting these *P^kn^* values versus the size of the minichromosomes revealed that the reduction of knot formation observed in the large minichromosomes (YCp50, YEp13, and YRp21) was overall significant (Fig 5E). Finally, as in the case of YEp13, inactivation of the Smc6/5 complex did not change DNA knotting probability in any of the constructs inspected (YRp4, 2‐micron plasmid, YCp50 and YRp21) (Fig 6A–E).

The above experiments corroborated that the Lk distribution of the different minichromosomes did not change upon inactivation of the SMCs, thereby excluding that *P^kn^* changes were consequent to alterations of DNA supercoiling or chromatin structure. The above results also evidenced that, before inactivation of the SMCs, the slope of *P^kn^* as minichromosomes increased in size (Figs 4F, 5E, 6E) was alike in all the strains. This similarity corroborated that the knot minimization mechanism is constitutive and robust. This mechanism is apparently sustained by the activity of condensin, since its inactivation restored the DNA length‐dependent entanglement of chromatin (Fig 4F).

## Discussion

The intrinsic capacity of topoisomerase II to simplify the equilibrium topology of DNA in free solution is commonly stated as the mechanism that prevents indiscriminate entanglement of intracellular DNA. This assumption, however, had never been experimentally tested until the present study. Our results show that disrupting the simplification activity of cellular topo II does not increase DNA knotting in chromatin. Apparently, the equilibrium topology of chromatinized DNA is not recognized by topo II in the same way as in free DNA. While these negative results cannot formally discard some role of the simplification capacity of topo II *in vivo*, the marked effects produced by condensin indicate that minimizing the entanglement of intracellular DNA mainly depends on this SMC complex.

### Mechanism of condensin to minimize DNA entanglements

Our finding that condensin minimizes the knotting probability of intracellular DNA seems a priori counterintuitive. Normally, any condition that folds or compacts DNA should promote its topological entanglement, not the opposite. Consistent with this notion, early *in vitro* studies found that condensin markedly increases topo II‐mediated knotting of DNA (Kimura et al, 1999; Losada & Hirano, 2001). DNA knotting and catenation were also found stimulated by cohesin (Losada & Hirano, 2001), the Smc5/6 complex (Kanno et al, 2015), and bacterial SMCs (Petrushenko et al, 2006; Bahng et al, 2016). These observations supported the notion that SMCs can embrace or bring in close proximity two or more segments of DNA. However, since SMCs had to be added in large molar excess (> 30:1) over circular DNA molecules to stimulate knotting or catenation, these experiments did not reflect a physiological context. Conversely, current evidence that individual condensin complexes can translocate along DNA (Terakawa et al, 2017) and produce the extrusion of DNA loops (Ganji et al, 2018) explain how condensin might promote the removal of DNA knots. Computational simulations of LE activity indicated that the extrusion process would tighten any intra‐ or inter‐molecular entanglement of DNA and enforce its removal by topo II (Goloborodko et al, 2016a; Racko et al, 2018; Orlandini et al, 2019). As a result, LE activity would reduce the equilibrium fractions of DNA links and knots, whereas LE inactivation would reestablish the equilibrium fractions (i.e., random entanglements of the DNA), which escalate proportionally to DNA length (Frank‐Kamenetskii et al, 1975; Rybenkov et al, 1993; Shaw & Wang, 1993). Remarkably, this prospect matches with the effects of condensin inactivation on minichromosomes of increasing size (Fig 4F).

**Figure 7:**
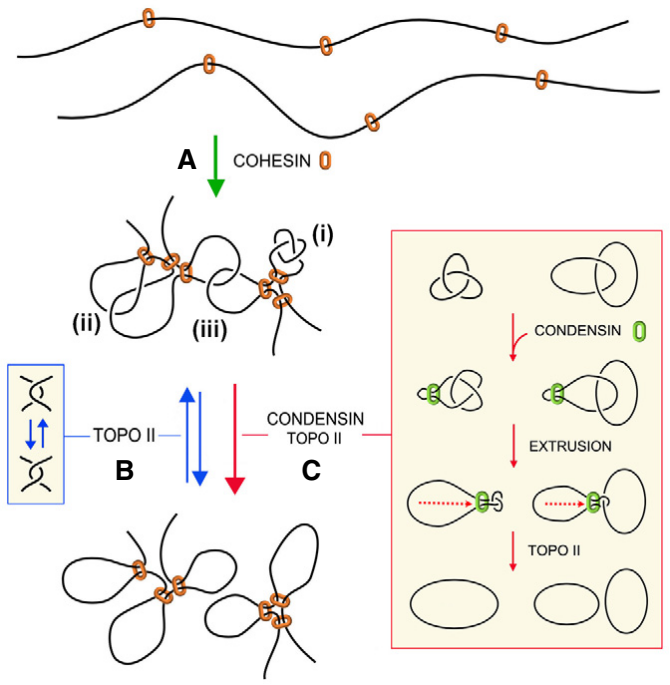
Model of condensin role in the minimization of DNA entanglements Cohesin generates and stabilizes DNA loops to organize interphase chromatin into topological domains.Random DNA strand passage activity of topo II can either remove or produce DNA entanglements within and across such topological domains. Juxtapositions of DNA segments within a loop can lead to the formation of knots (i), whereas juxtapositions of DNA segments belonging to nearby loops or adjacent domains can lead to the formation of intra‐ (ii) or inter‐molecular (iii) DNA interlinks.To minimize the occurrence of these entanglements, condensin might use its DNA loop extrusion activity to constrict intra‐ and inter‐molecular interlinks and so bias the DNA strand passage activity of topo II to remove them. This condensin function may operate during interphase to facilitate chromatin transactions and during cell division to enforce the removal of sister chromatid interlinks.

Since condensin minimizes intramolecular entanglements of DNA (knots), it might operate similarly to remove inter‐molecular DNA tangles such as the sister chromatid interlinks (SCI) that arise during DNA replication. A compaction‐independent role of condensin has been involved in the removal of these linkages (D'Amours et al, 2004; Renshaw et al, 2010). Moreover, although sister chromatids remain in very close proximity by the effect of cohesin until anaphase, the removal of SCI is nearly completed at the end of prophase (Nagasaka et al, 2016). However, inactivation of condensin during metaphase results in de novo formation of SCI (Sen et al, 2016; Piskadlo et al, 2017), which implies that condensin promotes the unlinking of sister chromatids while their close proximity still favors interlinking. In this respect, it was proposed that positive DNA supercoils generated by condensin in mitotic chromatin produce a bias in topo II function to remove the SCIs (Baxter et al, 2011; Sen et al, 2016). However, *in vitro* studies indicated that condensin does not compact DNA by inducing DNA supercoiling (Eeftens et al, 2017). Moreover, recent *in vivo* studies have shown that positive supercoiling of DNA markedly increases the formation of DNA knots (Valdes et al, 2019). Accordingly, if condensin were generating supercoils to promote the removal of SCI, that would in turn increase knot formation in mitotic chromatin. This prospect is inconsistent with our results, which show that condensin minimizes the occurrence of knots without altering DNA supercoiling both in interphase and mitotic chromatin. Therefore, our findings support the notion that the removal of intra‐ and inter‐molecular DNA entanglements could be promoted via the LE activity of condensin (Fig 7).

### Distinct effects of condensin and cohesin

In contrast to condensin, inactivation of cohesin and the smc5/6 complex did not increase knot formation. Moreover, cohesin inactivation slightly reduced *P^kn^* both in G1 and mitotic cells. This observation indicates that the plausible implication of cohesin on knot formation must be independent of its role in sister chromatid cohesion. In that case, the distinct effects of condensin and cohesin on *P^kn^* are striking since both complexes have LE activity (Davidson et al, 2019; Kim et al, 2019). Indeed, LE activity of cohesin *in vivo* accounts for the peaks and strikes observed in Hi‐C matrices, which are commonly translated as topological associated regions (TADs) in G1 cells (Fudenberg et al, 2016, Sanborn et al, 2015). Recent studies confirmed that cohesin‐mediated loops and the positions of TADs emerge quickly after telophase by producing contact patterns consistent with a LE process (Abramo et al, 2019; Zhang et al, 2019).

We can postulate several non‐excluding hypotheses to explain the different effects of condensin and cohesin on DNA knotting. One possibility could rely on the dynamics of their LE activity (Fig 7). Cohesin is likely to conduct discrete LE events to generate structural loops within specific boundaries. Subsequent stabilization of such loops would then favor intramolecular entanglement of DNA, as has been demonstrated with polymer simulations (Najafi & Potestio, 2015). Conversely, condensin may perform more dynamic rounds of LE without specific boundaries to scan the presence of DNA entanglements and promote their removal genome‐wide. This scenario might be analogous to that occurs in mitotic chromatin, where cohesin may favor SCI formation by maintaining sister chromatids in close proximity (Sen et al, 2016; Goloborodko et al, 2016b; Piskadlo et al, 2017), whereas condensin might be performing continuous rounds of LE to enforce the removal of SCI. A second possibility could rely on distinct coordination of condensin and cohesin with topo II activity. Based on immunofluorescence and ChIP data, topo II occupies similar genomic loci to condensin and cohesin, but their functional interplay remains unknown. Some studies suggested that condensin can physically interact with topo II and stimulate its activity (Bhat et al, 1996; D'Ambrosio et al, 2008a). Yet, other studies have failed to confirm a physical interaction (Bhalla et al, 2002; Lavoie et al, 2002; Cuvier & Hirano, 2003) or a stimulatory effect (Charbin et al, 2014). Likewise, a physical or functional interaction of cohesin and topo II has been proposed, as both complexes colocalize at DNA loop boundaries (Uuskula‐Reimand et al, 2016; Canela et al, 2017). Lastly, the distinct effects of condensin and cohesin could result from unequal binding to minichromosomes. This possibility, however, seems less likely considering the comparable abundance and broad chromosomal distribution of both complexes in budding yeast (Glynn et al, 2004; Wang et al, 2005). Accordingly, the effects of condensin and cohesin on *P^kn^* are accentuated with DNA length independently of the functional elements present in the minichromosomes. Only the lack of effects observed upon the inactivation of the Smc5/6 complex could be attributed to the lower abundance of this complex in comparison with cohesin and condensin (Aragon, 2018).

### Role of condensin during interphase

The generally established essential function of condensin is the compaction and individualization of sister chromatids to facilitate their segregation during cell divisions (Strunnikov et al, 1995; Hirano et al, 1997). To this end, condensin might play both an active role in promoting the removal of SCI (Sen et al, 2016; Piskadlo et al, 2017) and a structural role in organizing the axial architecture of mitotic chromosomes (Maeshima & Laemmli, 2003; Ono et al, 2003; Walther et al, 2018). These mitotic roles are achieved by the single condensin complex found in yeast cells and by the two condensin complexes (condensin I and II) found in metazoans (Hirota et al, 2004; Hirano, 2012). However, former studies in budding yeast revealed that condensin is also present in interphase chromatin (Freeman et al, 2000; Lavoie et al, 2002), where it is distributed over the length of every chromosome throughout the cell cycle (Wang et al, 2005; D'Ambrosio et al, 2008b). Likewise, condensin II is also present in interphase chromatin in metazoans (Hirano, 2012; Frosi & Haering, 2015). The role of condensin during interphase is unknown, but its inactivation causes large‐scale changes in the chromatin structure of budding yeast (Bhalla et al, 2002; Lazar‐Stefanita et al, 2017; Paul et al, 2018). Inactivation of condensin II produces intermixing of chromosomal territories in Drosophila (Rosin et al, 2018; Rowley et al, 2019) and an increase of inter‐chromosome associations in mammals (Nishide & Hirano, 2014). Other studies concur that condensin disruption alters a wide range of processes including gene regulation, DNA repair and recombination (Frosi & Haering, 2015; Paul et al, 2019). It is intriguing how so many functions and phenotypes are connected to condensin activity. According to our findings, the answer could be that condensin is promoting the removal of harmful DNA knots and interlinks that topo II activity might produce during topological equilibration of chromatin fibers and domains (Fig 7). Such DNA entanglements can alter, for instance, the progression of RNA polymerases and the assembly of nucleosomes, as demonstrated by *in vitro* studies (Portugal & Rodriguez‐Campos, 1996; Rodriguez‐Campos, 1996). Therefore, the failure of condensin to promote DNA untangling is expected to interfere with multiple genome transactions during interphase, in addition to the individualization of chromosomes during cell division.

The unanticipated role of condensin in minimizing DNA entanglements raises new questions, such as how the LE activities of condensin and cohesin may interplay with each other throughout the cell cycle. A similar issue arises in mitotic chromatin, in which the cohesion of sister chromatids, the removal of SCI, and DNA looping along the axial architecture of chromosomes involve the coordination of distinct SMC activities. Another relevant matter is the interplay of SMCs and type‐2 topoisomerases, which are highly conserved from bacteria to eukaryotes. The coordination of these two essential machineries might have been primordial throughout evolution to minimize DNA entanglements as genomes increased in size and complexity.

## Materials and Methods

### DNA constructs and yeast strains

Plasmids YEp13, YEp24, YRp21, YCp50, YRp5, YRp4, and YRp3 (Appendix Fig S1) were amplified in E*scherichia coli* and, when indicated, converted into circular minichromosomes by transforming *Saccharomyces cerevisiae* using standard procedures (Valdes et al, 2018). Cellular topo II assays were done in the topo I‐deficient strains *JCW27* (*M*A*Ta*, *∆top1*, *his3‐*D*200*, *leu2‐*D*1*, *trp1‐*D*63*, *ura3–52*) and *JCW28* (*MATa*, *∆top1*, *top2‐4*, *his3‐D200*, *leu2‐D1*, *trp1‐D63*, *ura3–52*) (Trigueros & Roca, 2001). Condensin function was tested in *AS330* (*MATa*, *smc2‐8*, *ura3*, *leu2*, *lys2*, *his3*, *ade2*) (Freeman et al, 2000). The *smc2‐8* mutation was introduced in yeast strains *JCW25* (*MATa*, *his3‐*D*200*, *leu2‐*D*1*, *trp1‐*D*63*, *ura3–52*) and its derivative *JCW26* (*top2‐4*) by two‐step gene replacement involving the counter selectable marker *URA3* (Rothstein, 1991). Cohesin function was tested in the strain *K5832* (*MATa*, *scc1‐73*, *ade2‐1*, *ura3–52*, *TRP+*, *can1‐100*, *leu2‐3*, *112*, *his3‐11*) (Michaelis et al, 1997). Smc5/6 function was tested in *CCG1428* (*MATa*, *smc6‐9*, *bar1∆*, *leu2‐3 112*, *ura3‐52*, *his3‐D200*, *trp1‐D63*, *ade2‐1*, *lys2‐801*, *pep4)* (Torres‐Rosell et al, 2005). Thermo‐sensitivity of SMC complexes and topoisomerase mutants was corroborated by drop growth assays (Appendix Fig S2).

### Topo II activity in crude yeast lysates

To target cellular topo II activity with ICRF‐193 (Sigma‐Aldrich), *JCW27 (∆top1)* cells bearing YEp13 were grown at 30°C in synthetic dropout ‐LEU media containing 2% glucose. Exponential 50 ml cultures (OD_600_ = 0.6–0.8) were harvested and washed twice in TE (Tris–HCl 10 mM (pH 8) EDTA 1 mM) and resuspended at 4°C in 1 ml of lysis buffer (Tris–HCl 10 mM pH 8.0, EDTA 1 mM, EGTA 1 mM, NaCl 150 mM, DTT 1mM, Triton X‐100 0.1%, pepstatin 1µg/ml, leupeptin 1µg/ml, PMSF 1 mM). Resuspended cells were transferred to 15‐ml conic tubes and mixed with 1 ml of acid‐washed glass beads (425–600 µm, Sigma). Mechanic lysis of> 80% cells was achieved by stirring six times with a vortex apparatus for 30 sec at 4°C. Glass beads and large cell debris were removed by centrifugation (2000 g x 2 min at 4°C). Cell lysates (0.5 ml) were supplemented with 5 mM MgCl_2_ and 2 mM ATP and with 100 ng of a negatively supercoiled control plasmid (YEp24). Following incubation at 30°C for 20 min, ICRF‐193 was added (100 μM) and incubation continued at 30°C for 10 min. Reactions were quenched by adding EDTA (20 mM) and SDS (0.2%) and extracted twice with phenol and once with chloroform. Nucleic acids were precipitated with ethanol and dissolved in 100 µl of TE containing RNAse‐A. Following 10‐min incubation at 37°C, ammonium acetate was added to 0.5 M and DNA was precipitated with ethanol. Each DNA sample was dissolved in 40 µl of TE prior gel electrophoresis. To test *Top2‐∆83* activity in yeast, *JCW28 (∆top1 top2‐4)* cells bearing YEp13 and the expression plasmids *pGAL1T2 or pGAL1T2Δ83* (Martinez‐Garcia et al, 2014) were grown at 26°C in synthetic dropout ‐URA ‐LEU media containing 2% glucose. Exponentially growing cultures were diluted to OD_600_ = 0.1 in YEP containing 2% raffinose. When OD_600_ = 0.6‐0.8 was reached at 26°C, galactose was added to a 2% final concentration and the cell cultures were shifted to 35°C for 2 h. Cells were harvested and crude lysates were prepared by stirring with glass beads as described above. A sample of the lysates was loaded in SDS–PAGE gels to confirm the extrachromosomal expression of *TOP2* and *Top2‐∆83* proteins. Upon addition of 5 mM MgCl_2_, 2 mM ATP and 100 ng of negatively supercoiled plasmid YEp24, the lysates were incubated for 30 min at 35°C. Reactions were quenched and nucleic acids isolated for gel electrophoresis analyses as described above.

### SMC mutants culture and DNA extraction

Yeast strains bearing distinct circular minichromosomes were grown at 26°C in the adequate synthetic dropout media supplemented with 2% glucose. Exponentially growing cultures OD_600_ = 0.6–0.8 were maintained at 26°C or shifted to 35°C for 60 min to inactivate the temperature‐sensitive alleles. To arrest the cells in G1, alpha‐factor to a final concentration of 2 mg/L was added to exponentially growing cultures every 30 min for 2 h at 26°C and then for one additional hour upon shifting one half of the cultures to 35°C. To arrest the cells in metaphase, nocodazole was added to exponentially growing cultures to a final concentration of 15 mg/mL for 2 h at 26°C and then for one additional hour upon shifting one half of the cultures to 35°C. Following the inactivation of the temperature‐sensitive alleles, the DNA topology of circular minichromosomes was fixed *in vivo* by quickly mixing the liquid cultures with one cold volume (−20°C) of ETol solution (Ethanol 95%, 28 mM Toluene, 20 mM Tris–HCl pH 8.8, 5 mM EDTA) (Diaz‐Ingelmo et al, 2015). To measure cellular DNA content (1n, 2n), about 10^6^ of ETol fixed cells were washed with saline‐sodium citrate (SSC) buffer, incubated for 1 h at 37°C in SSC containing 0.1 mg/mL RNase‐A and again incubated for 1 h at 50°C in SSC containing 1 mg/mL Proteinase K. Cell samples in 1 mL SSC were sonicated for two 30 sec cycles at 4C and incubated at 25°C for 1h in presence of 3 mg/mL propidium iodide prior flow cytometry reading on a Gallios (Beckman Coulter) cell analyzer. To extract total DNA, ETol fixed cells from 25 ml cultures were sedimented, washed twice with TE, resuspended in 400µl of TE, and transferred to a 1.5‐ml microfuge tube containing 400µl of phenol and 400µl of acid‐washed glass beads (425–600 µm, Sigma). Mechanic lysis of> 80% cells was achieved by shaking the tubes in a FastPrep® apparatus for 10 sec at power 5. The aqueous phase of the cell lysates was collected, extracted with chloroform, precipitated with ethanol, and dissolved in 100 µl of TE containing RNAse‐A. Following 10‐min incubation at 37°C, ammonium acetate was added to 0.5 M and DNA was precipitated with ethanol. Each DNA sample was dissolved in 40 µl of TE prior gel electrophoresis.

### DNA electrophoresis for topology analyses

Lk distributions of control plasmid YEp24 were examined with 1D‐electrophoreses carried out in 0.8% agarose gels in TBE buffer (89 mM Tris‐borate, 2 mM EDTA) plus 0.2 µg/ml of chloroquine and run at 50V for 14 h. Lk distribution of minichromosomes YEp13, YRp21, and YCp50 were examined with 2D‐electrophoreses carried out in 0.6% agarose gels (20 × 20 cm) in TBE buffer plus 0.6 µg/ml of chloroquine in the first dimension (30V for 36 h) and in TBE buffer plus 3 µg/ml of chloroquine in the second dimension (80V for 4 h). 2D electrophoreses of YRp3, YRp4, YRp5, and 2‐micron circles were carried out in 0.8% agarose gels (20 × 20 cm) in TBE buffer plus 0.6 µg/ml of chloroquine in the first dimension (50V for 14 h) and TBE buffer plus 3 µg/ml of chloroquine in the second dimension (60V for 6 h).

To examine the DNA knots formed in the minichromosomes, DNA samples were nicked with endonuclease BstNBI (NEB). 2D‐electrophoreses of nicked DNA of YRp3, YRp4, and YRp5 circles were carried out in a 0.9% agarose gel (20x20 cm) in TBE buffer at 33V for 40 h in the first dimension and at 150V for 3 h in the second dimension. 2D‐electrophoreses of nicked DNA of 2‐micron and YCp50 circles were carried out in a 0.6% agarose in TBE buffer at 25V for 40 h in the first dimension and at 125V for 4 h in the second dimension. 2D‐electrophoreses of nicked DNA of YEp13 and YRp21 circles were carried out in a 0.4% agarose in TBE buffer at 25V for 40 h in the first dimension and at 125V for 4 h in the second dimension.

All 2D‐gels were blot‐transferred to positively charged nylon membranes (Hybond‐N^+^, Amersham Biosciences). Blots were hybridized with minichromosome DNA probes labeled with AlkPhos Direct (GE Healthcare®). Probe signals were visualized following incubation with CDP‐Star detection reagent (GE Healthcare®) for 10 min at room temperature and recorded on X‐ray films. DNA knot probability (*P^Kn^*) was calculated as described previously (Valdes et al, 2018) by quantifying non‐saturated signals obtained with serial dilutions of DNA knot samples or with different exposure periods, using the ImageJ software. *P^Kn^* values are the relative abundance of total knot populations with respect to the total amount of unknotted and knotted DNA circles.

## Author contributions

Experiments: JR, SD, BM‐G, AV; Data analysis: JR, SD; Manuscript writing: JR, SD, AV.

## Conflict of interest

The authors declare that they have no conflict of interest.

## Supporting information



Appendix

Expanded View Figures PDF

Review Process File
