# Supplementary material for: Condensin minimizes topoisomerase II‐mediated entanglements of DNA in vivo
Source: EMBO J. 2020 Nov 6;40(1):e105393. doi: 10.15252/embj.2020105393 (PMC7780148; doi:10.15252/embj.2020105393)
Supplement: Supplementary file 1 — Appendix [file EMBJ-40-e105393-s001.pdf]

Condensin minimizes topoisomerase II-mediated entanglements of DNA *in vivo*

Sílvia Dyson, Joana Segura, Belén Martínez-García, Antonio Valdés, and Joaquim Roca*

Appendix

Figure S1. Plasmids and minichromosomes analyzed in the study.

Figure S2. Drop assays of yeast strains used in the study.

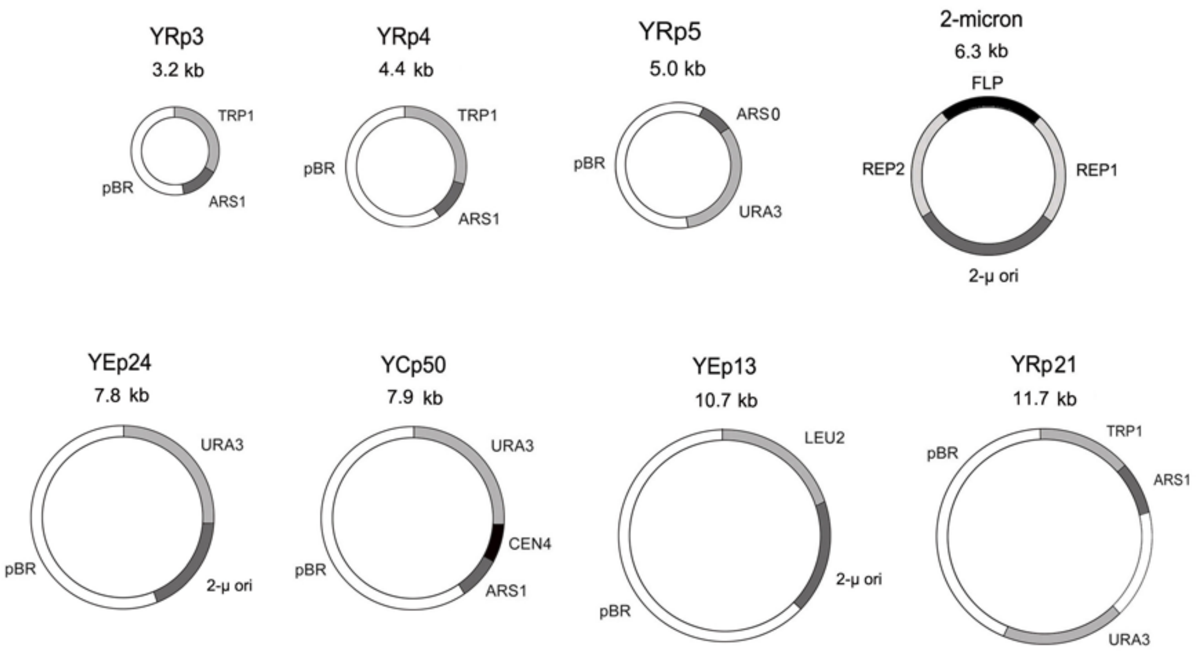

Appendix Figure S1. Plasmids and minichromosomes analyzed in the study.

Minichromosomes containing pBR sequences were amplified as bacterial plasmids in *Escherichia coli* and used to transform *Saccharomyces cerevisiae*. The 2-micron plasmid was endogenous in all the yeast strains used. Minichromosomes differed in size, replication origins (autonomous replication sequences (ARS) or 2-micron origin), presence of centromere (CEN), and selectable gene markers (TRP1, URA3, LEU2) as indicated.

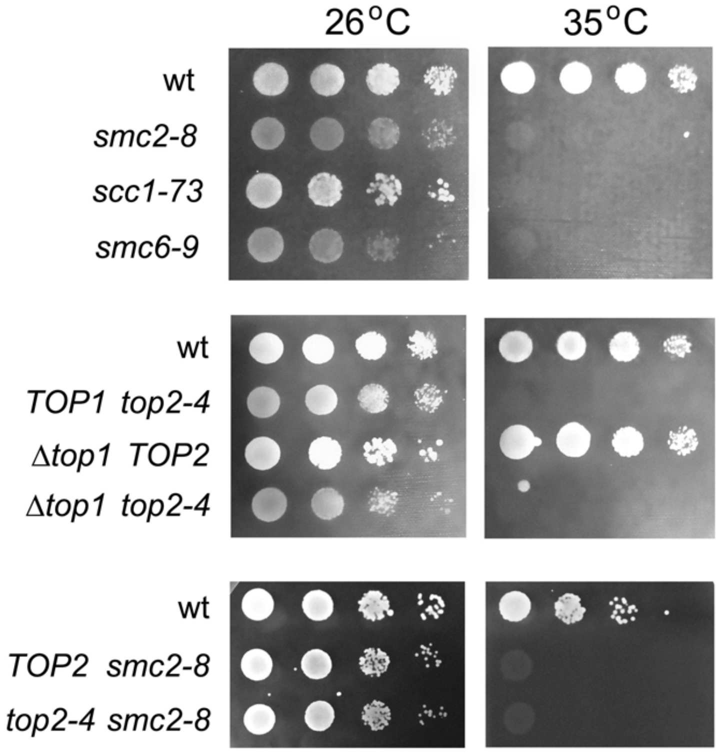

Appendix Figure S2. Drop assays of yeast strains used in the study.

Yeast strains carrying thermo-sensitive mutations that inactivate SMC complexes and/or topoisomerase activities were grown to late-log phase at 26°C and spotted in 10-fold dilutions on YPD. Plates were incubated at 26°C or 35°C. *JCW25* was used as the wild-type (wt) control in all plates.
