# Supplementary material for: Condensin minimizes topoisomerase II‐mediated entanglements of DNA in vivo
Source: EMBO J. 2020 Nov 6;40(1):e105393. doi: 10.15252/embj.2020105393 (PMC7780148; doi:10.15252/embj.2020105393)
Supplement: Supplementary file 2 — Expanded View Figures PDF [file EMBJ-40-e105393-s002.pdf]

# Expanded View Figures

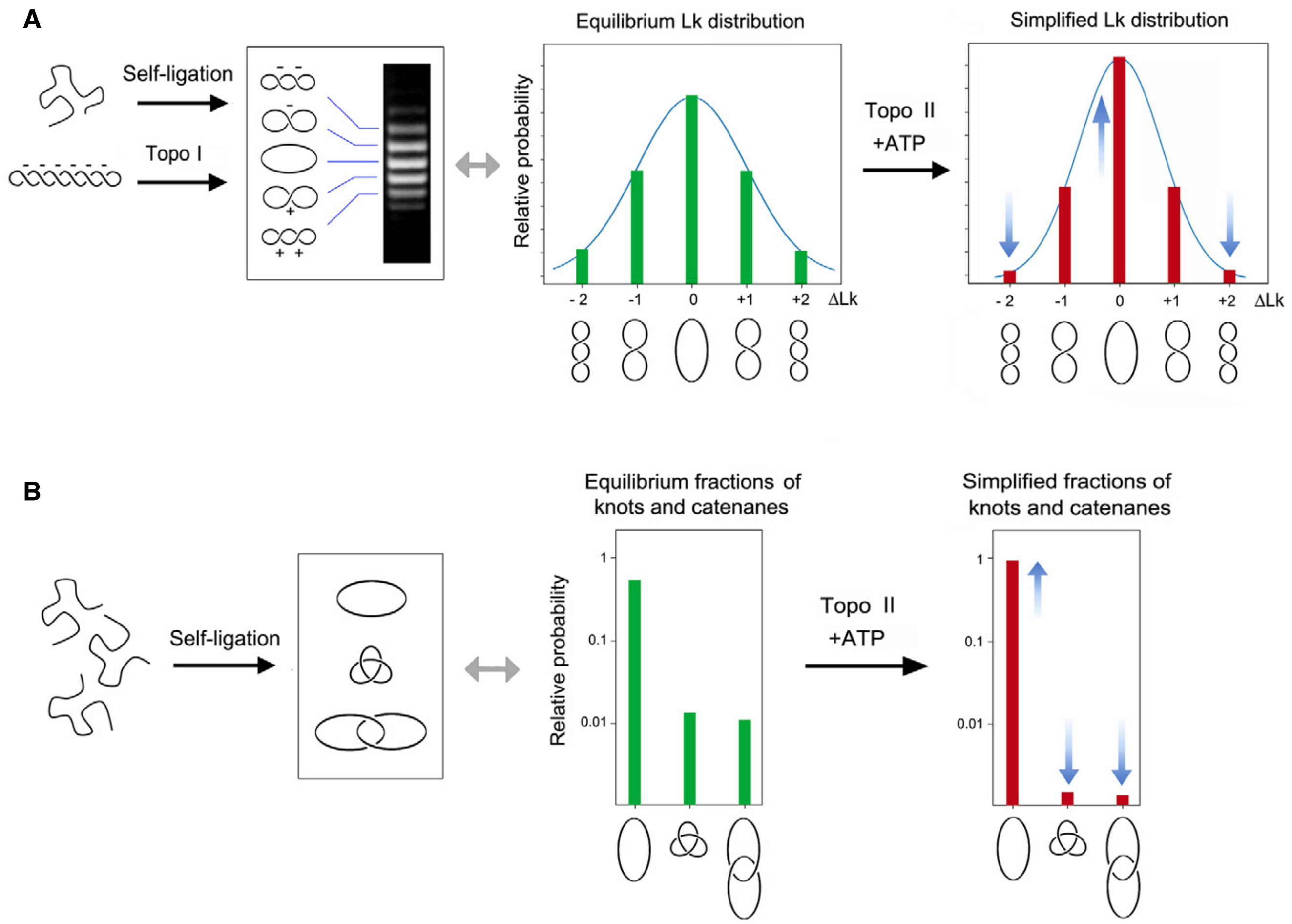


**Figure EV1. Equilibrium DNA topology and its simplification by Topo II.**

A Either the self ligation of a linear DNA duplex into a covalently closed ring or the relaxation of a DNA plasmid with a type-1B topoisomerase (topo I) produce identical equilibrium distributions of Lk topoisomers, which reflect the thermal fluctuations (twisting and bending) of DNA molecules in free solution. ATP-dependent DNA passage catalyzed by topo II simplifies (i.e., reduces the variance, narrows) the equilibrium distribution of Lk topoisomers. ΔLk values indicate the Lk difference relative to the distribution center (Lk = 0).

B Circularization of linear DNA molecules in free solution can also produce knotted and/or catenated DNA rings. Knotting probability increases with DNA length, whereas catenane probability increases with DNA concentration. As in the case of the Lk distribution, the knotting and catenation probability reflect the equilibrium topology of DNA in free solution. ATP-dependent DNA passage catalyzed by topo II markedly reduces (i.e., simplifies) the equilibrium fractions of knotted and catenated forms.

© 2020 The Authors

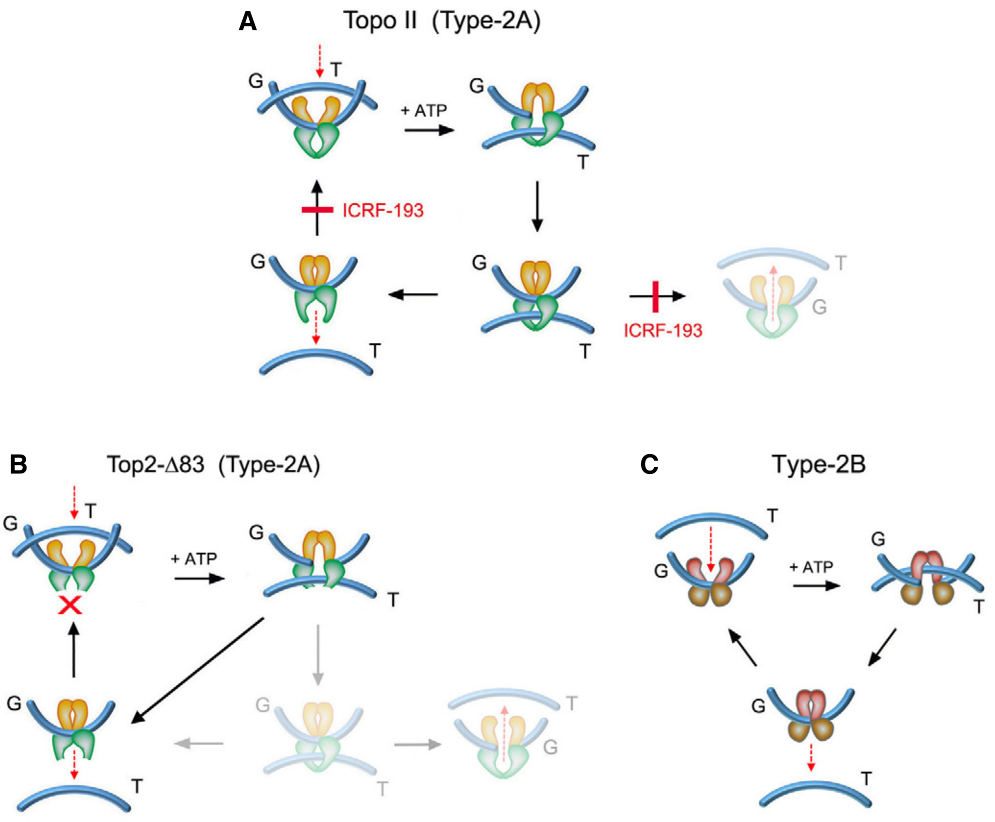


**Figure EV2. Conditions that preclude topo II capacity to simplify DNA topology.**

A The topo II inhibitor ICRF-193 impedes the reopening of the N-gate once the T-segment has been captured and passed across the G-segment. ICRF-193 blocks thereby the enzyme turnover and the plausible backtracking of the T-segment across the G-segment. When topo II activity is quenched with ICRF-193, the last DNA-passage event conducted by the enzyme does not simplify the equilibrium DNA topology.

B The topo II construct top2-Δ83, in which the C-gate has been deleted, is able to perform DNA passage and the T-segment cannot backtrack since it is freed upon crossing the G-segment. This truncated enzyme can relax and unlink DNA molecules but has lost the capacity to simplify the equilibrium DNA topology.

C Type-2B topoisomerases are mechanistically similar to type-2A topoisomerases (topo II). The T-segment is captured by the N-gate and is passed across the bended G-segment at the DNA-gate. However, type-2B topoisomerases do not have a C-gate, so the passed T-segment is naturally freed upon crossing the G-segment. As in the case of top2-Δ83, type-2B topoisomerases relax and unlink DNA molecules but do not simplify equilibrium DNA topology.

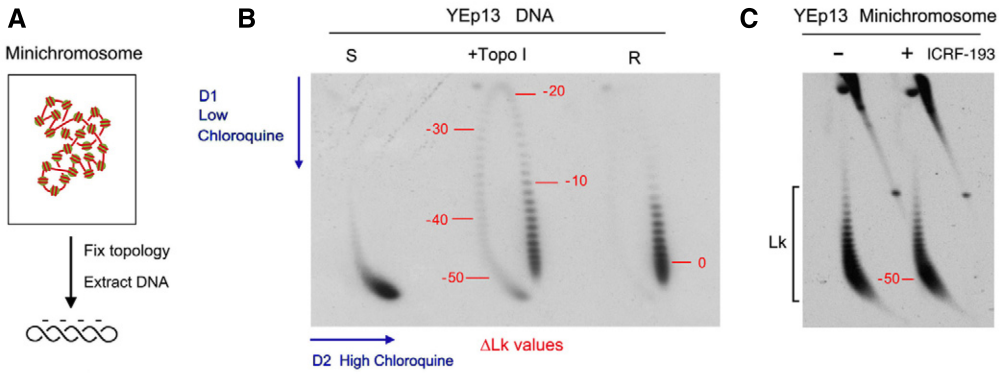


**Figure EV3.**

 © 2020 The Authors

**Figure EV3. 2D gel electrophoresis of the DNA linking number distribution of circular minichromosomes.**

A DNA molecules extracted from yeast circular minichromosomes are negatively supercoiled since each nucleosome constrains about one negative supercoil ($\Delta Lk \approx -1$).

B 2D gel electrophoresis of covalently closed DNA circles, in which the first and second gel dimensions are run in the presence of low and high concentrations of chloroquine, respectively, allow the Lk distribution of DNA topoisomers to be resolved along an arch, in which Lk values increase clockwise. The 2D gel shows highly negatively supercoiled (S), partially relaxed (+topo I), and fully relaxed (R) forms of the YEp13 plasmid. Numbers in red indicate approximate ΔLk values relative to the center of the relaxed (R) Lk distribution ($\Delta Lk = 0$).

C 2D gel electrophoresis of DNA of the YEp13 minichromosome (as in Fig 2C). Comparison of the gels in (B and C) indicates that DNA in the YEp13 minichromosome is negatively supercoiled and has ΔLk close to −50. This value is consistent with the plausible number of nucleosomes assembled in YEp13 (10.7 Kb). However, note that since the outline of Lk distributions can vary in separate 2D gels (i.e., due to differences in tank dimensions, power supply and temperature during electrophoresis), only DNA samples that ran in the same gel (side by side) can be accurately compared.

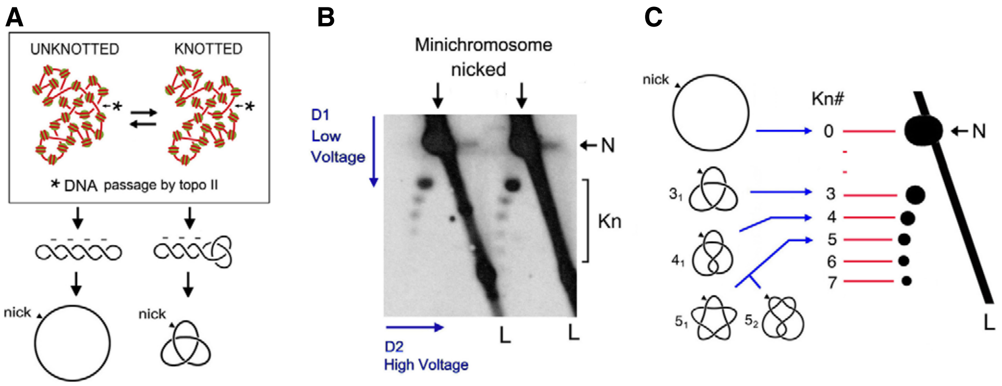


**Figure EV4. 2D gel electrophoresis of DNA knots formed in yeast circular minichromosomes.**

A DNA molecules extracted from yeast minichromosomes might contain knots due to the knotting-unknotting activity of intracellular topo II. Knotted and unknotted molecules are hard to distinguish when DNA is supercoiled because all of them present similar compaction. Upon nicking the DNA, supercoiling is dissipated and knotted molecules remain more compact than unknotted ones.

B 2D gel electrophoresis of nicked DNA of the YEp13 minichromosome (as in Fig 2C). The first and second gel-dimensions run at low and high voltage, respectively. In the first dimension, knotted molecules (Kn) are more compacted and so move faster than unknotted ones (N). In the second gel-dimension, knotted molecules are retarded from the diagonal of linear DNA fragments (L), which produces a strong signal due to genomic DNA present in the samples.

C Identification of DNA knot populations according to the irreducible number of DNA crossings of each knot (Kn#). From the position of the unknotted circle that has zero crossings (0), a ladder of knot populations of increasing complexity begins with the knot of three crossings ($3_1$), followed by the knot with four crossing ($4_1$), two knots with five crossings ($5_1$ and $5_2$), and so on.

© 2020 The Authors

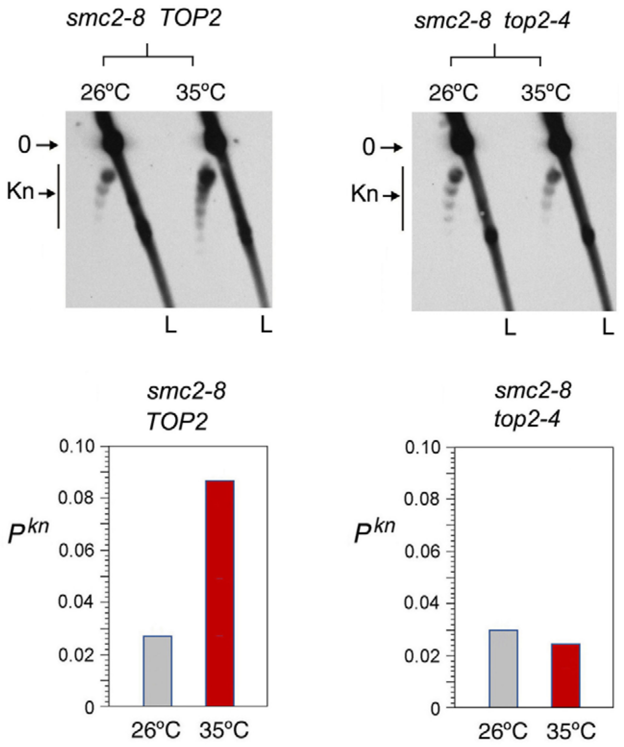


**Figure EV5. Topo II dependence of knot formation upon inactivation of condensin.**

The *smc2-8* mutation was introduced by gene replacement in yeast strains JCW25 (*TOP2*) and JCW26 (*top2-4*). The 2D gel electrophoresis shows the knotted forms of the minichromosome YEp13 in the resulting *smc2-8 TOP2* and *smc2-8 top2-4* mutants sampled at 26°C and after shifting the cell cultures to 35°C for 60 min. Gel signals: 0, unknotted DNA circles; Kn, knotted forms. L, linear DNA fragments. Graphs: $P^{kn}$ of YEp13 before and after inactivation of the thermo-sensitive alleles.


© 2020 The Authors
